# Considerations for treatment duration in responders to immune checkpoint inhibitors

**DOI:** 10.1136/jitc-2020-001901

**Published:** 2021-03-02

**Authors:** Thomas U Marron, Aideen E Ryan, Sangeetha M Reddy, Sabina Kaczanowska, Rania H Younis, Dipti Thakkar, Jiajia Zhang, Todd Bartkowiak, Rachel Howard, Kristin G Anderson, Daniel Olson, Abdul Rafeh Naqash, Ravi B Patel, Esha Sachdev, Maria E Rodriguez-Ruiz, Michal Sheffer, Sarah Church, Christopher Fuhrman, Abigail Overacre-Delgoffe, Rosa Nguyen, Vaia Florou, Jessica E Thaxton, David H Aggen, Jennifer L Guerriero

**Affiliations:** 1 Department of Medicine, Division of Hematology Oncology, Mount Sinai School of Medicine, New York, New York, USA; 2 Discipline of Pharmacology & Therapeutics, Lambe Institute for Translational Medicine, National University of Ireland, Galway, Ireland; 3 The University of Texas Southwestern Medical Center, Dallas, Texas, USA; 4 Pediatric Oncology Branch, Center for Cancer Research, National Cancer Institute, National Institutes of Health, Bethesda, Maryland, USA; 5 Department of Oncology and Diagnostic Sciences, School of Dentistry, and the Tumor Immunology and Immunotherapy Division UMGBCCC, University of Maryland, Baltimore, Maryland, USA; 6 Department of Oral Pathology, Faculty of Dentistry, Alexandria University, Alexandria, Egypt; 7 Department of Pharmacology, Hummingbird Bioscience, Singapore; 8 Bloomberg Kimmel Institute for Cancer Immunotherapy, Johns Hopkins, Baltimore, Maryland, USA; 9 Department of Cell and Developmental Biology, Vanderbilt University, Nashville, Tennessee, USA; 10 Health Informatics, H Lee Moffitt Cancer Center and Research Institute, Tampa, Florida, USA; 11 Clinical Research Division, Fred Hutchinson Cancer Research Center, Seattle, Washington, USA; 12 Department of Immunology, University of Washington, Seattle, WA, USA; 13 Department of Medicine, The University of Chicago Comprehensive Cancer Center, Chicago, Illinois, USA; 14 Division of Cancer Treatment And Diagnosis, National Cancer Institute, Bethesda, Maryland, USA; 15 Department of Radiation Oncology, University of Pittsburgh Hillman Cancer Center, Pittsburgh, Pennsylvania, USA; 16 Department of Medicine, Division of Oncology, University of Southern California, Los Angeles, California, USA; 17 Radiation Oncology, Clinica Universidad de Navarra Departamento de Oncologia Medica, Pamplona, Spain; 18 Department of Medical Oncology, Dana-Farber Cancer Institute, Boston, Massachusetts, USA; 19 Transitional Sciences, NanoString Technologies Inc, Seattle, Washington, USA; 20 Department of Immunology, Children’s Hospital of Pittsburgh of University of Pittsburgh Medical Center, University of Pittsburgh, Pittsburgh, Pennsylvania, USA; 21 Pediatric Oncology Branch, National Cancer Institute, Bethesda, Maryland, USA; 22 Internal Medicine, Huntsman Cancer Institute, Salt Lake City, Utah, USA; 23 Department of Orthopedics and Physical Medicine, Medical University of South Carolina, Charleston, South Carolina, USA; 24 Department of Microbiology and Immunology, Medical University of South Carolina, Charleston, SC, USA; 25 Hollings Cancer Center, Charleston, SC, USA; 26 Genitourinary Oncology Service, Department of Medicine, Memorial Sloan Kettering Cancer Center, New York, New York, USA; 27 Department of Surgery, Division of Breast Surgery, Brigham and Women's Hospital, Boston, Massachusetts, USA; 28 Breast Tumor Immunology Laboratory, Dana-Farber Cancer Institute, Boston, Massachusetts, USA

**Keywords:** Costimulatory and Inhibitory T-Cell Receptors, Immunotherapy, Review, Melanoma

## Abstract

Immune checkpoint inhibitors (ICIs) have improved overall survival for cancer patients, however, optimal duration of ICI therapy has yet to be defined. Given ICIs were first used to treat patients with metastatic melanoma, a condition that at the time was incurable, little attention was initially paid to how much therapy would be needed for a durable response. As the early immunotherapy trials have matured past 10 years, a significant per cent of patients have demonstrated durable responses; it is now time to determine whether patients have been overtreated, and if durable remissions can still be achieved with less therapy, limiting the physical and financial toxicity associated with years of treatment. Well-designed trials are needed to identify optimal duration of therapy, and to define biomarkers to predict who would benefit from shorter courses of immunotherapy. Here, we outline key questions related to health, financial and societal toxicities of over treating with ICI and present four unique clinical trials aimed at exposing criteria for early cessation of ICI. Taken together, there is a serious liability to overtreating patients with ICI and future work is warranted to determine when it is safe to stop ICI.

## Durable responses to cancer immunotherapy

Cancer care across histologies has changed at break-neck speed in the past decade. Since the first Food and Drug Administration (FDA) approval of the Cytotoxic T-lymphocyte Antigen-4 (CTLA-4) antibody ipilimumab for metastatic melanoma a decade ago, dozens of approvals for antibodies targeting programmed cell death protein 1 (PD-1) or programmed death ligand 1 (PD-L1) alone or in combination with ipilimumab and/or chemotherapy have followed. Historically in oncology, with few exceptions, 5-year survival has been associated with ‘cure’. In the last few years—specifically in melanoma where the majority of immune checkpoint inhibitor (ICI) agents were first tested in a phase 3 setting—mature 5-year overall survival (OS) data is now being reported. In the KEYNOTE-001 clinical trial testing the safety and efficacy of pembrolizumab (anti-PD-1), there was a reported 5-year OS of 34% in all patients, with 16% of patients achieving a complete response (CR).^1^ In KEYNOTE-001, discontinuation was permitted in patients after receiving ≥2 pembrolizumab doses beyond the initial determination of CR and who received pembrolizumab treatment for ≥6 months. CR was confirmed by imaging scans ≥4 weeks apart and discontinuation was at the discretion of the investigator and if the patient desired. These patients were eligible to receive a second course of pembrolizumab. Seventy-two patients met criteria to discontinue therapy per protocol and entered observation. Sixty-seven achieved CR and five achieved partial response (PR) as best overall response (BOR) while seven of these patients had progressive disease post cessation (six prior CR, one prior PR). Strikingly, however, 90% of responses were maintained.^1^ Similarly, durable responses were seen in the KEYNOTE-006 phase 3 trial, investigating single-agent treatment of pembrolizumab or ipilimumab in 834 advanced melanoma patients.^2^ Nineteen per cent of patients completed the planned 2 years of pembrolizumab treatment, of which 20% achieved a BOR of CR, 67% PR and 13% had stable disease (SD).^3^ Responses continued after treatment cessation in 76% of CRs, 77% of PRs and 54% of SD. Remarkably, 8% of patients with previous BOR of PRs converted to CRs after cessation of pembrolizumab.^3^ Among patients who received 2 years of treatment of pembrolizumab and had at least SD, 78% of patients exhibited sustained disease control and remained progression free 2 years after pembrolizumab completion. The 2-year OS was 96% and the 3-year OS was 94%. Estimated 2-year progression free survival (PFS) was 85% for CR, 82% for PR and 40% for SD. Importantly, 23 patients with CRs who stopped pembrolizumab treatment earlier than 2 years, as allowed by the protocol, exhibited a PFS rate of 86%, similar to CRs who completed the full 2-year regimen. As one may expect, at the end of 2 years, patients with SD progressed more quickly than those with CR or PR. Of the patients taken off pembrolizumab, 74% remained progression free, while 26% had progressive disease. Of those patients that progressed, 44% received a second course of pembrolizumab, and more than half were again able to achieve a response.^3^ Further evidence for stable responses in patients with metastatic melanoma after early discontinuation of ICI was shown in an analysis of a real-world cohort. Of 52 patients who electively discontinued PD-1 inhibitors after 1 year (>6 months and <18 months) in the setting of ongoing treatment response or disease stability, after median follow-up of 20.5 months (range 3–49.2) from treatment discontinuation, 39 (75%) patients remained without disease progression (median PFS not reached).^4^

While treatment discontinuation due to adverse events makes outcome comparison challenging, evidence from the Keynote-006 trial suggests that some patients may continue to derive benefit from ICI therapy after discontinuation, suggesting durable benefits may be feasible from shorter courses of therapy than defined by current protocols. In a pooled analysis of randomized phase 1 and phase 2 trials of combination nivolumab and ipilimumab, efficacy outcomes were found to be comparable between patients who discontinued treatment due to immune-related adverse events (irAEs) during the early phase of the trial, and those who did not.^5^ Here, the proportion of CRs, and the time to response, were approximately equal in the discontinued group and the continuation group. On reanalysis of the same data, Horiguchi *et al* further concluded that patients in the discontinuation group were in fact predicted to live longer than those in the continued treatment group, lending credence to the notion that patients experiencing irAEs during immunotherapy may be those in which a strong immune response has been induced.^6^ Similarly, long-term responses to ipilimumab can be achieved after discontinuation due to irAE even after short treatment durations.^7^

Evidence from these early pembrolizumab trials in melanoma reflects data from nivolumab and combination nivolumab–ipilimumab trials,^8^ as well as real-world data on patients who cease therapy due to toxicity or patient preference. These data demonstrate that patients can experience durable responses with low incidence of relapse after significantly shorter treatment times than are mandated by trial design.^9–11^ The likelihood of an individual patient experiencing a sustained response after a relatively short time on treatment is likely to depend on several factors. While biomarkers to identify patients who will achieve a durable response are lacking, there are significant data demonstrating a correlation between depth and duration of response. In one real-world analysis of patients who discontinued therapy in the absence of disease progression or treatment limiting toxicity, 14% of CRs experienced progressive disease during follow-up, as compared with 32% and 50% of partial responders and patients with SD, respectively.^12^ Another single institution series observed that among 102 patients that achieved CR to anti-PD-1 therapy who discontinued treatment after a median treatment time of 9.4 months, 72% remained alive at 3-year follow-up without further treatment.^13^ Smaller studies have provided further anecdotal evidence of this pattern, with partial responders experiencing longer PFS after treatment discontinuation than patients with SD.^14 15^ Collectively, this suggests that among complete responders, risk of relapse after discontinuation is low even after treatment for only 6 months, though this data also demonstrate that a significant number of patients who achieve only radiographic PR or even SD may derive long-term benefit from shorter periods to treatment. Studies specifically designed to investigate duration of therapy, and biomarkers of durable responses are required to establish optimal treatment durations for those patients with PR or SD.

As data from trials across histologies mature, and with increased real-world experience, clinicians and patients achieving prolonged benefit from ICI are increasingly being faced with the dilemma of whether or not to proceed according to the design of trials that led to FDA approval, as has been the standard of care, or to risk discontinuing a successful therapy. Based on the collective experience with maintenance chemotherapy, and our understanding that metastatic cancer is nearly always a terminal illness, early trials in melanoma which specified either 2 years or indefinite therapy were followed by a large number of registrational studies in a variety of other cancers (table 1). These trials have perpetuated what is now considered a standard trial design of prolonged maintenance therapy, despite the data from melanoma trials suggesting that this may constitute overtreatment. Indeed, while early trials treated indefinitely, and the majority of trials today treat for 2 years, the benefit of ICI is typically seen very early, potentially even within the first week.^16^ These neoadjuvant trials in which patients have received relatively brief courses of therapy ahead of surgery have countered the belief that response to immunotherapy is slow, though radiographic responses may be delayed due to inability to differentiate a robust immune response (and subsequent radiographic scar formation) from progressive disease. If there is a vaccinal effect on lymphoid memory, one could hypothesize that only short treatments are needed, akin to the comparatively brief treatments needed with IL-2 to induce durable remissions.^17^ However, one retrospective analysis of a large cohort of patients who had achieved a CR did find an association between recurrence and ICI treatment of less than 6 months.^12^ In summary, early data from retrospective cohorts and pooled/subgroup analysis from clinical trials suggest that certain subsets of patients, particularly patients with durable response or irAEs, might benefit from cessation of immunotherapy, yet additional work will be needed for clinical utility. Prospective studies with elective discontinuation design are warranted to further elucidate the timing and indication for immunotherapy discontinuation, and additionally trials must assess the impact on OS—specifically addressing whether it is safe, and potentially non-inferior, to hold therapy and restart if/when a patient’s disease progresses.

**Table 1:** Approvals for the use of immune checkpoint inhibitors

Target	Agent	Indication	FDA approval based on registrational trial
PD-1	Pembrolizumab	Colorectal cancer—microsatellite instable	Until progression, unacceptable toxicity, or up to 24 months
		Cutaneous squamous cell carcinoma	Until progression, unacceptable toxicity, or up to 24 months
		Endometrial carcinoma*	Until progression, unacceptable toxicity, or up to 24 months
		Esophageal carcinoma	Until progression, unacceptable toxicity, or up to 24 months
		Gastric carcinoma	Until progression, unacceptable toxicity, or up to 24 months
		Head and neck squamous cell carcinoma*	Until progression, unacceptable toxicity, or up to 24 months
		Hepatocellular carcinoma	Until progression, unacceptable toxicity, or up to 24 months
		Hodgkin’s lymphoma	Until progression, unacceptable toxicity, or up to 24 months
		Malignant pleural mesothelioma	Until progression, unacceptable toxicity, or up to 24 months
		Melanoma†	Until progression or unacceptable toxicity
		Merkel cell carcinoma	Until progression, unacceptable toxicity, or up to 24 months
		Microsatellite instable cancers (histology agnostic)	Until progression, unacceptable toxicity, or up to 24 months
		Non-small cell lung carcinoma*	Until progression, unacceptable toxicity, or up to 24 months
		Primary mediastinal B-cell lymphoma	Until progression, unacceptable toxicity, or up to 24 months
		Renal cell carcinoma*	Until progression, unacceptable toxicity, or up to 24 months
		Small cell lung carcinoma	Until progression, unacceptable toxicity, or up to 24 months
		Urothelial carcinoma	Until progression, unacceptable toxicity, or up to 24 months
	Nivolumab	Colorectal cancer - microsatellite instable*	Until progression or unacceptable toxicity
		Esophageal carcinoma	Until progression or unacceptable toxicity
		Head and neck squamous cell carcinoma*	Until progression or unacceptable toxicity
		Hepatocellular carcinoma*	Until progression or unacceptable toxicity
		Hodgkin’s lymphoma	Until progression or unacceptable toxicity
		Melanoma*†	Until progression or unacceptable toxicity
		Malignant pleural mesothelioma	Until progression, unacceptable toxicity, or up to 24 months
		Non-small cell lung carcinoma*	Until progression or unacceptable toxicity
		Renal cell carcinoma*	Until progression or unacceptable toxicity
		Small cell lung carcinoma*	Until progression or unacceptable toxicity
		Urothelial carcinoma	Until progression or unacceptable toxicity
	Cemiplimab	Cutaneous squamous cell carcinoma	Until progression or unacceptable toxicity
PD-L1	Atezolizumab	Breast cancer, triple negative*	Until progression or unacceptable toxicity
		Hepatocellular carcinoma*	Until progression or unacceptable toxicity
		Melanoma*	Until progression or unacceptable toxicity
		Non-small cell lung carcinoma*	Until progression or unacceptable toxicity
		Small cell lung carcinoma*	Until progression or unacceptable toxicity
		Urothelial carcinoma	Until progression or unacceptable toxicity
	Durvalumab	Non-small cell lung carcinoma (Stage III)	Following chemoradiation, until progression, unacceptable toxicity, or up to 12 months
		Small cell lung carcinoma*	Until progression or unacceptable toxicity
		Urothelial carcinoma	Until progression or unacceptable toxicity
	Avelumab	Gestational trophoblastic neoplasia	Until progression or unacceptable toxicity, of for three cycles after normalization of hCG
		Merkel cell carcinoma	Until progression or unacceptable toxicity
		Renal cell carcinoma*	Until progression or unacceptable toxicity
		Urothelial carcinoma	Until progression or unacceptable toxicity

*Approval includes combination therapies.

†Approval also exists in the adjuvant setting for 12 months.

FDA, Food and Drug Administration; PD-1, programmed cell death protein 1; PD-L1, programmed death ligand 1.

### Physical toxicity from prolonged immunotherapy

**Figure 1:**
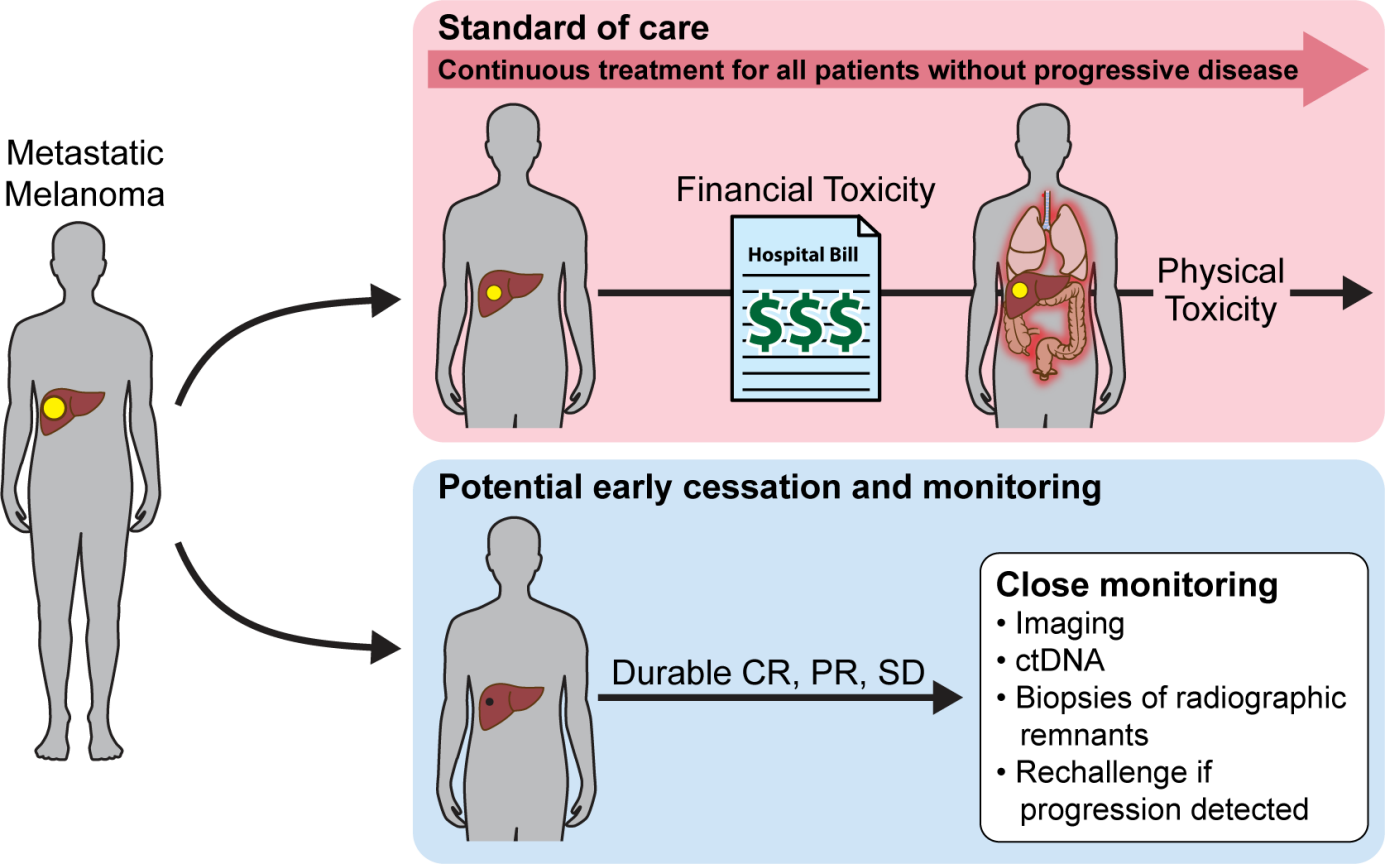
Patients receiving immune checkpoint inhibitors without progressive disease are treated for an undefined period, which can extend several years and may impose both financial and physical toxicity. Clinical trials are needed to determine criteria that would allow potential early cessation and monitoring thus eliminating both financial and physical toxicities. CR, complete response; ctDNA, circulating tumor DNA; PR, partial response; SD, stable disease.

Immunotherapy treatments generally have reduced high-grade toxicities compared with conventional cytotoxic chemotherapy.^2 18^ Despite this, it is not uncommon for patients to experience irAEs, most frequently involving the skin, gastrointestinal tract, lung and endocrine glands but also potentially manifest as neurologic, hepatic, rheumatological, renal and cardiac toxicities.^19 20^ Although most irAEs are of mild or moderate severity (grades 1 and 2), clinically significant grade 3 or 4 irAEs have been reported in up to 20% of patients with single-agent PD-1 immunotherapy.^20^ Grade 3 or 4 irAEs are even more prevalent in patients treated with combination treatment targeting both PD-1 and CTLA-4, affecting 59% of patients.^21^ These side effects frequently result in interruptions of immunotherapy treatment and require immunosuppressants such as corticosteroids which themselves carry the risk of toxicities and may be associated with poorer survival outcomes when used long-term.^22^ Biological immunomodulatory agents may be required to manage patients with severe or life threatening irAEs, which can result in permanent treatment discontinuation and/or significant and long-term patient morbidity, if not death (figure 1).^23^

Due to the heterogeneous nature and time to onset of these toxicities, early identification of irAE symptoms and timely intervention can be challenging. Although a majority of irAEs occur within the first 5–15 weeks, there are reports of late-onset toxicities both in the setting of ongoing immunotherapy and after treatment cessation.^24–27^ These delayed irAEs have been reported to occur months to years after discontinuation of therapy.^28 29^ In a recent study, approximately half of the delayed irAE cases were observed among patients who had previously reported on-treatment irAEs, which in many cases were the reason for cessation of therapy.^29^ However, this study found only 21 cases of delayed irAE in response to checkpoint blockade immunotherapy from a decade of literature, suggesting a low prevalence of serious irAEs after discontinuation of therapy.^29^ While these delayed irAE are more rare than early-onset toxicities they do occur and can be severe; in one small retrospective study looking at 325 patients treated with ICI, within the 12% of patients continuing therapy for more than 1 year, 15% developed delayed irAEs.^30^ Case reports periodically highlight late onset irAEs, yet in the absence of a consistent reporting system, knowledge-based decisions on optimal duration of checkpoint inhibitor therapy in responsive patients is challenging.^28^ Regardless, it’s important to note that prolonged use of ICI comes with physical risk.

### Financial toxicity from prolonged treatment with immunotherapy

Although ICIs have undoubtedly made an impact on the survival of patients, they have also imposed a significant financial burden on patients, their families, as well the public and private healthcare industry. As a consequence of durable remissions with limited understanding of the optimal duration of ICI treatment, patients often remain on immunotherapy for prolonged periods of time (up to several years). This has a significant financial impact on both the patient and the healthcare system (figure 1).

A recent National Cancer Institute study based on retrospective Surveillance, Epidemiology and End Results registry data estimated the annual expense of cancer care in the United States to be over US$200 billion in 2020, and projected that this will approach US$250 billion by 2030.^31^ Given the rapid pace of development of novel cancer therapies, these projections may significantly underestimate true societal expenses. While the prolonged survival—and potential cure—that is being realized with cancer immunotherapy is a phenomenal step forward for the field of oncology, the rapid rise in expense must be promptly addressed to ensure continued access to these agents, the development of new immunotherapies to further improve on the current state of cancer care, and continued scientific progress.

Along with the societal financial toxicity, the increase in drug costs encourages for-profit insurance providers to place greater financial responsibility on the patient in the form of increased deductibles, copays and premiums, making the associated personal and societal financial toxicities unavoidable.^32^ While cancer-associated costs will be specific to each patient depending on the diagnosis, treatment type and level of insurance coverage, many patients report the need for assistance in budgeting, understanding their coverage, and seeking financial help due to nuances of personalized therapy.

With treatment costs for cancer immunotherapy often in excess of US$100 000 per year, even those with a standard employer-sponsored health plan with 20% coinsurance are faced with bills that exceed 50% of the average US household income. The combination of nivolumab and ipilimumab for a typical patient has been estimated to cost US$295 566, an out-of-pocket cost of US$60 000 annually.^33^ As many as 40% of patients have been found to experience difficulties paying medical bills, with up to 11% missing recommended treatments, 12% lowering the dose of prescription medications, and 12% missing additional appointments or follow-up testing to reduce overall costs.^34^ In addition to the implications for treatment discontinuation or refusal, cancer patients are over 2.5 times more likely to declare bankruptcy as compared with healthy adults,^35^ and financial difficulties related to cancer care are a risk factor for mortality.^36^ The out-of-pocket cost of cancer immunotherapy to the individual patient, as well as the cost that long-term treatment exacts on a family and caregivers due to missed work and lost income, serves as additional motivation for identifying the optimal duration of treatment.

### Trials investigating cessation of ICI therapy

**Figure 2:**
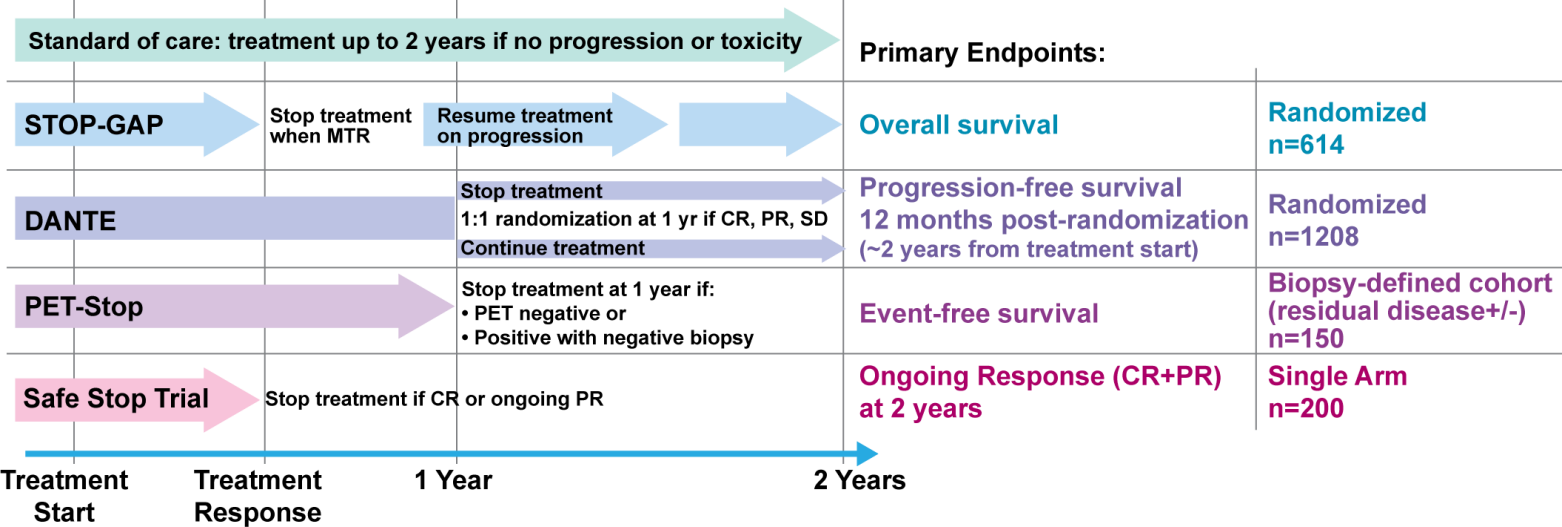
Ongoing prospective clinical trials to determine criteria for early discontinuation of immune checkpoint inhibitors. CR, complete response; MTR, maximal tumor response; PR, partial response; SD, stable disease.

Registrational phase 3 trials have been insufficiently powered to determine an adequate duration to maintain ICI therapy, nor the potential safety hazards of relinquishing treatment. A single industry-sponsored trial in non-small-cell lung cancer (NSCLC) has evaluated the potential to stop immunotherapy early, and confirmed the need for continued treatment, though the design of the trial to include patients without mandating SD and durable disease response has drawn criticism.^37^ With significant data on long-term follow-up from the initial ICI trials, melanoma is the ideal clinical space to test whether to stop treatment early. In the past year, several trials have opened, all sponsored by government backed healthcare organizations, which are aimed to define optimal duration (figure 2). The DANTE (Detection And screening of early lung cancer with Novel imaging TEchnology) trial (ISRCTN15837212), for example, is a randomized phase 3 trial designed to evaluate feasibility of stopping first-line anti-PD-1 monotherapy (nivolumab or pembrolizumab) at 12 months in patients who are progression-free. Led by the UK National Cancer Research Institute Skin Cancer Clinical Studies Group, DANTE randomizes patients with unresectable stage III or stage IV melanoma who have received 1 year of PD-1 blockade without having their disease progress to either (1) stopping treatment (with the option to restart anti-PD-1 therapy or commence other treatment on progression) or (2) continuing treatment for another year or until disease progression/unacceptable toxicity. This is a non-inferiority trial that will enroll 1208 patients, and the primary outcome is PFS 1 year from randomization (2 years from initiation of immunotherapy), while secondary outcomes include quality of life assessment, OS, response rate and both physical and financial toxicities. Its notable that the investigators are also assessing cost-effectiveness, and they are also using multiple standardized quality of life metrics including the validated quality of life questionnaires (QLQs) European Organization for Research and Treatment of Cancer (EORTC) QLQ-C30, QLQ-MEL38 and the EQ-5D-5L questionnaires for up to 18 months following randomization; while the trial is defined to assess non-inferiority in terms of PFS, effects on various physical and financial aspects affecting quality of life are potentially of equal importance.

Other trials are exploring stopping at even earlier time points based on treatment response without a minimum prespecified treatment duration, and also looking at ability to subsequently rechallenge. The Safe Stop trial (NTR7502, EudraCT: 2018-001384-23) is a study in the Netherlands looking at early discontinuation of first line anti-PD-1 therapy (nivolumab or pembrolizumab) for advanced or metastatic melanoma patients who have achieved a confirmed CR (with an interval confirmatory imaging of at least 6 weeks after first documentation) or an ongoing PR (with an interval of 12 weeks after first documentation). This single arm study will prospectively assess rates of ongoing responses in 200 patients at 2 years as its primary endpoint, with secondary endpoints evaluating duration of response, PFS, rate of anti-PD-1 rechallenge on progression and associated response and survival metrics, and OS. Associated studies (Safe Stop-QoL) will also measure quality of life, patient work productivity and impact on caregivers, which will help address key survivorship questions for this population in which many patients are considered cured of their disease. Like Safe Stop, the Canadian Clinical Trials Group study STOP-GAP (NCT02821013) is assessing the potential to stop treatment after maximal tumor response (MTR), which is determined by at least two radiologic measurements 3 months apart. A total of 614 patients with unresectable stage III or stage IV melanoma are being randomized 1:1 in this phase 3 trial to either standard 2 years of therapy in the absence of disease progression, or treatment until MTR with retreatment at the time of progression. The primary endpoint is OS, with secondary endpoints measuring PFS, objective response rate, AE rate, health-related quality of life and economic analysis. Notably, STOP-GAP is unique as compared with DANTE or Safe Stop in that its principal emphasis is on the role of re-challenge and impact of a ‘stop and go’ as opposed to continuous approach on OS, rather than the specific question of optimal initial duration of treatment.

There is a growing appreciation that imaging criteria for response as used in DANTE, Safe Stop and STOP-GAP only partially capture pathologic response, as some patients have persistent lesions radiographically without viable cancer. PET-Stop (EA6192; NCT04462406) is an ECOG/ACRIN cooperative group-led study that will address this in its biomarker-driven trial on early discontinuation of anti-PD-1 therapy in stage IIIB and stage IV melanoma. After 1 year of immune checkpoint blockade (pembrolizumab or nivolumab±ipilimumab), patients will receive a positron emission tomography (PET) scan to qualify for trial enrolment. Patients will have their immunotherapy held if their PET scan is negative or if it is positive but with subsequent negative biopsy of remaining hypermetabolic lesions (Arm A). The primary endpoint for this trial will assess event free survival after 1 year on study (ie, 2 years from starting ICI). Patients enrolled who have a positive PET scan with a biopsy showing viable cancer or inability to perform biopsy will enroll onto Arm B and be followed with serial imaging and repeat biopsy at conclusion of addition 1 year of anti-PD-1 therapy. Secondary endpoints include conversion of Arm B to pathological CR, OS, extended duration of therapy beyond 2 years total and toxicities.

Given the heterogeneity of both tumor and host biology, future strategies will need to explore a personalized treatment approach. It will be integral to obtain adequate tumor biopsies and optimal the correct blood samples in clinical trials to achieve deeper understanding of patient-specific tumor molecular features, baseline intratumoral immune and stromal environment, and both local and systemic immune responses will be critical to identifying patients likely to respond to therapy and maintain a durable response, as well as those who may be pre-disposed to toxicity. The translational tissue collection in PET-Stop will serve as an important resource for these exploratory investigations. Tumor tissue and peripheral blood collections are planned for this purpose as part of PET-Stop; gene expression in baseline tumor biopsies will characterize the preliminary immune infiltration, and serial blood analysis of circulating tumor DNA (ctDNA) and lymphoid and myeloid composition by mass cytometry (cytometry by time of flight; CyTOF) will be performed to assess for predictive and/or prognostic utility. Given emerging data on the utility of ctDNA to identify melanoma patients with deep responses to targeted and immunotherapy,^38–40^ it is critical to use this biomarker, potentially in addition to immune signatures in the peripheral blood in prospective trials, to confirm the predictive and prognostic utility of these technologies.

To our knowledge, early cessation of ICI is only currently being prospectively investigated in in the unresectable/metastatic melanoma setting, due to the high rates of durable response seen in these patients and extensive long-term follow-up from early trials in melanoma. Given the expanded use of ICI in the neoadjuvant/adjuvant setting—where the needed treatment period is also undefined^41–43^—and maturing 5-year data on the use of ICI in other histologies such as NSCLC and renal cell carcinoma,^44–46^ where there are also durable responses seen, many subsequent trials will be needed in these alternate settings to define optimal duration of therapy, in particular given the astronomical cost associated with these therapies being given to patients, some of whom may not even need adjuvant therapy.^47^ Future trials should continue to build off this framework to ultimately lead biomarker driven trials that can establish in which patients we can safely discontinue ICI therapy to spare the patient, and the healthcare system, significant toxicities.
